# Enhancing the Biodegradability, Water Solubility, and Thermal Properties of Polyvinyl Alcohol through Natural Polymer Blending: An Approach toward Sustainable Polymer Applications

**DOI:** 10.3390/polym16152141

**Published:** 2024-07-27

**Authors:** Abdallah S. Elgharbawy, Abdel-Ghaffar M. El Demerdash, Wagih A. Sadik, Mosaad A. Kasaby, Ahmed H. Lotfy, Ahmed I. Osman

**Affiliations:** 1 Materials Science Department, Institute of Graduate Studies and Research (IGSR), Alexandria University, 163 Horrya Avenue, P.O. Box 832, Shatby, Alexandria 21526, Egypt; ab_pet_88@hotmail.com (A.S.E.);; 2 The Egyptian Ethylene and Derivatives Company (Ethydco), Alexandria 21544, Egypt; 3 School of Chemistry and Chemical Engineering, Queen’s University Belfast, Belfast BT9 5AG, UK

**Keywords:** biodegradable polymers, polyvinyl alcohol, corn starch, hydroxypropyl methylcellulose, polymer blending, mechanical properties

## Abstract

The escalating environmental crisis posed by single-use plastics underscores the urgent need for sustainable alternatives. This study provides an approach to introduce biodegradable polymer blends by blending synthetic polyvinyl alcohol (PVA) with natural polymers—corn starch (CS) and hydroxypropyl methylcellulose (HPMC)—to address this challenge. Through a comprehensive analysis, including of the structure, mechanical strength, water solubility, biodegradability, and thermal properties, we investigated the enhanced performance of PVA-CS and PVA-HPMC blends over conventional polymers. Scanning electron microscopy (SEM) findings of pure PVA and its blends were studied, and we found a complete homogeneity between the PVA and both types of natural polymers in the case of a high concentration of PVA, whereas at lower concentration of PVA, some granules of CS and HMPC appear in the SEM. Blending corn starch (CS) with PVA significantly boosts its biodegradability in soil environments, since adding starch of 50 *w*/*w* duplicates the rate of PVA biodegradation. Incorporating hydroxypropyl methylcellulose (HPMC) with PVA not only improves water solubility but also enhances biodegradation rates, as the addition of HPMC increases the biodegradation of pure PVA from 10 to 100% and raises the water solubility from 80 to 100%, highlighting the significant acceleration of the biodegradation process and water solubility caused by HPMC addition, making these blends suitable for a wide range of applications, from packaging and agricultural films to biomedical engineering. The thermal properties of pure PVA and its blends with natural were studied using diffraction scanning calorimetry (DSC). It is found that the glass transition temperature (Tg) increases after adding natural polymers to PVA, referring to an improvement in the molecular weight and intermolecular interactions between blend molecules. Moreover, the amorphous structure of natural polymers makes the melting temperature ™ lessen after adding natural polymer, so the blends require lower temperature to remelt and be recycled again. For the mechanical properties, both types of natural polymer decrease the tensile strength and elongation at break, which overall weakens the mechanical properties of PVA. Our findings offer a promising pathway for the development of environmentally friendly polymers that do not compromise on performance, marking a significant step forward in polymer science’s contribution to sustainability. This work presents detailed experimental and theoretical insights into novel polymerization methods and the utilization of biological strategies for advanced material design.

## 1. Introduction

PVA is an organic compound that is commonly present in odorless and tasteless powders and particles. Its steady chemical characteristics, hydrophilicity, and biocompatibility are excellent. The main method for creating PVA is to hydrolyze polyvinyl acetate and replace the acetate group with a hydroxyl group [1]. Controlling the hydrolysis stage allows PVA with different amounts of hydrolysis to be produced [2].

Since PVA is an expensive polymer with a slow rate of biodegradation, researchers have focused on improving its properties during the past 10 years by combining it with different kinds and quantities of other biopolymers that are acceptable to the environment. All of the used polymers and biopolymers were considered; however, due to their molecular structures and the presence of -OH functional groups, chitosan, carboxymethyl cellulose (CMC), and starch were given greater attention when it came to mixing them with PVA films [3].

As a hydrophilic polymer, starch is semi-crystalline in nature. It is one of the biopolymers that has been studied the most for use in food packaging because of its affordability, nontoxicity, high biodegradability, and accessibility [4]. The molecular structure of starch is that of a complex branched polymer with (1–4) linkages connecting the D-glucose units and branch sites. Because it is renewable, biodegradable, and easily obtainable, starch is one of the numerous natural resources that could be utilized to make biodegradable polymers [5,6]. Thermoplastic starch can be made by heating starch with the appropriate plasticizer. However, films produced completely from starch are not suitable for packing due to their brittleness, stiffness, and poor mechanical and thermal properties [7].

The term “hydroxypropyl methylcellulose” (HPMC) describes a type of cellulose ethers where one or more of the three hydroxyl groups present in the cellulose ring have been replaced [8]. With a wide range of applications in medicine administration, skin care products, adhesives, glue coatings, agriculture, and textiles, HPMC is a hydrophilic (water soluble), biodegradable, and biocompatible polymer [9]. Due to HPMC’s exceptional biocompatibility and minimal toxicity, scientists and scholars are interested in its potential applications in the biomedical field [10].

Microplastics are created as a result of the environmental degradation that occurs in polymeric materials when they are discharged into the environment. As long as they are in the environment, it is thought that these microplastics will increase the overall amount of microplastics in the ecosystem and pose a further ecological risk to the biota. The biota is in great danger of chemical and physical harm; the more microplastics there are, the longer they have been present in the environment [11]. Around 39,000 to 52,000 microplastic particles are consumed by humans each year alone through food and drink. Plastics carry chemicals that have been adsorbed to them as they enter the human food chain, additives from the manufacture process, and potentially even bacteria or parasites [12].

Cano et al. [13] prepared a PVA film with varying starch percentages (10–50%) and discovered that an increase in starch content affected the mechanical qualities, crystallinity level, and absorption of water of PVA/starch film, and vice versa. The control starch film’s mechanical characteristics improved as the PVA ratio was raised. However, the optical characteristics did not appreciably alter while the film was being stored. Gomez-Aldapa et al. [14] found that the incorporation of PVA considerably enhanced the mechanical qualities, gas permeability, and functional characteristics of the potato starch films. Furthermore, a combination of 60% PVA and 40% starch exhibited the best water vapor barrier, lower density, and solubility, as well as higher mechanical performance. Films made of potato starch and PVA had the right properties for possible applications in food packaging and as a biodegradable alternative for the replacement of synthetic packaging materials.

Negim et al. [15] created blended films of polyvinyl alcohol (PVA) starch (S) with glacial acetic acid added as a crosslinking agent and examined the characteristics of the blended films’ biodegradation. Burying PVA/S blend films in soil allowed researchers to study how they broke down biologically. The results showed that PVA/S mix films deteriorated in dry soil in 10–14 days, depending upon the molecular weight (Mw) of PVA. PVA blend films (205,000 g/mol) took 13–14 days to disintegrate, but PVA blend films (31,000 g/mol)/S only took 10 days. It is clear, for example, that PVA/S mix films biodegrade more slowly in dry soil when the molecular weight of PVA increases. In contrast, all PVA/S blends cause the blend films to deteriorate in a moist environment in less than three days. These results demonstrate that adding S to PVA enhances the polymer matrix’s starch-mediated degradation. 

Song et al. [16] developed starch/PVA films by using a blowing extrusion technique. The resulting film, with an St/PVA ratio of 4:6, showed excellent mechanical properties (tensile strengths of 21.0 MPa and elongation at break of 213.9%), cold-water solubility (dissolution time of 90 s), and oxygen barrier performance (oxygen transmission rate of 1.41 cm^3^/(m^2^ daybar)). The dissolved St/PVA films containing amphiphilic groups promoted the emulsification of butachlor, a fat-soluble liquid pesticide, and the dispersibility of oxyfluorfen, a fat-soluble solid pesticide. Additionally, a mechanism of interaction between the pesticide and the leaf surface of the weed was proposed to explain why pesticides wrapped in St/PVA films were more effective. It is successful in producing starch-based films suitable for pesticide inner packaging materials by employing PVA blending and MA-enhanced esterification, respectively.

Wei et al. [17] created starch/polyvinyl alcohol (PVA) degradable straws with different PVA concentrations using a twin-screw extrusion technique. The data showed that the starch/PVA straws with 40% PVA (PS4) had the maximum starch and PVA dispersion homogeneity to produce the best compatibility and that the compatibility size was below the micron level. The greatest texture densities were achieved by the strongest molecular interactions that were made possible by the highest hydrogen bonds between 40% polyvinyl alcohol and starch. Because of this, PS4′s mechanical properties and resistance to water were significantly improved by the strongest molecular bonds. When comparing the swelling volume of PS4 to that of the starch/PVA straw containing 0% PVA (PS0), the differences were 45.5% (4 °C) and 65.2% (70 °C). Following soaking, the diameter strength increased by 540.1% (4 °C for 1 h) and 638.7% (15 min) at 70 °C. Over 30-minute periods at 4 °C and 70 °C, respectively, there was a 45.3% and 27.6% drop in water absorption. Laoasoke et al. [18] created thin films with up to 20% cannabidiol (CBD) using hydroxypropyl methylcellulose (HPMC). Polyvinyl alcohol (PVA), which is pliable and soft, served as the supporting layer. The bilayer film had remarkable mechanical properties, with a 30% elongation and a tensile strength of 8.54 N/mm^2^. Adding a PVA backing layer improves the mechanical properties and can prolong the film disintegration process by up to 90 min. These results indicate that the HPMC-PVA bilayer is a suitable polymer matrix for cannabidiol delivery since it provides mucoadhesive properties for oral administration and controls release for the enhanced absorption of cannabidiol.

Huang et al. [19] examined the effects of a film based on roselle anthocyanin, polyvinyl alcohol, and hydroxypropyl methylcellulose (PHR) on the biochemical and flavor profiles of shrimp. Better preservation behaviors were observed with PHR films, and the film with the highest anthocyanin content also showed the best biochemical properties. In summary, the PHR film showed promise for extending the shelf life and changing the flavor characteristics of chilled shrimp.

Yu et al. [20] investigated the improvement in the mechanical and functional properties of chitosan/polyvinyl alcohol/hydroxypropyl methylcellulose/alizarin composite films by adding tea polyphenols and cinnamon essential oil. Their inclusion in composite films improved their elongation at break and strengthened their antioxidant and antibacterial properties. The water vapor permeability, water solubility, and water content of the composite films decreased, and their transparency was increased by the cinnamon essential oil. Excellent Ph responsiveness and UV barrier performance were demonstrated by the composite films containing additives. The formation of hydrogen bonds between the polymer molecules and additives was verified by X-ray diffraction and transform infrared spectroscopy. A better surface and cross-sectional morphology of the films was shown by the scanning electron microscope–focused ion beam findings. According to thermogravimetric and differential scanning calorimetry studies, the addition of cinnamon essential oil to the composite films resulted in improved thermal stability. Chen et al. [21] improved the mechanical and antibacterial properties of the polyvinyl alcohol/hydroxypropyl methylcellulose/chitosan mix film (CHP) by combining bamboo fiber with an agent. Following examinations with various coupling agent concentrations, the film produced with 1 mL of coupling agent performed the best overall. The film’s capacity to resist water was enhanced by a 369.3% reduction in water vapor permeability (WVP) and a 41.63% decrease in water solubility. The elongation at break increased by 8%, while the tensile strength increased by 61.97%. It is possible to create bioactive packaging using a composite film consisting of chitosan, hydroxypropyl methylcellulose, and polyvinyl alcohol that has been changed by a coupling agent and bamboo fiber, as this study shows.

**Figure 1:**
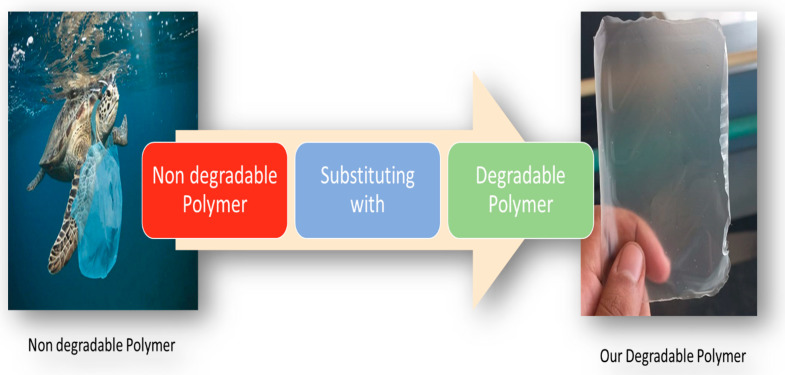
This study aims to substitute some applications of single-use polymers with an eco-friendly polymer.

In this paper, we are going to reveal the procedure of producing two polymer blends (corn starch/PVA and HPMC/PVA) and adding PVA in the same quantity to both polymers (starch/HPMC) to compare the differences that occur between the different blended films to determine the optimum blending ratio that gives the best properties of each blend, and we also investigate their properties, such as their mechanical, thermal, structural, and degradation properties. Eventually, we propose many applications for the two new blends that can replace single-use polymers. Figure 1 outlines the aim of this work of producing new degradable polymers that can substitute non-degradable single-use polymers in some applications, so that they can save living beings from the harmful effects of non-degradable polymers.

## 2. Materials and Methods

### 2.1. Materials

Polyvinyl alcohol with a molecular weight (Mw) of approximately 115,000 g/mol and a degree of hydrolysis (mole%) of 98–99 was purchased from Oxford Lab Fine Chem, India, with a purity of 99%. PVA was used as the main polymer to which the natural polymers added to form the required blends. Analytical-grade corn starch (Cs) with a purity of 99% was purchased from EL-Gomhoria Chemicals in Egypt and used as a polymer mixed with a varying quantity of PVA to form corn starch/PVA blend films. Hydroxypropyl methylcellulose (HPMC) was purchased in powder form from Parchem Fine and Specialty Chemical, New York, with a purity of 99%. HPMC polymer was blended with a different quantity of PVA to form HPMC/PVA blend films. Glycerol was purchased in liquid form from EL-Gomhoria Chemicals with a purity of 99% and used as a plasticizing agent to enhance the gelatinization of the different blended films. Polyethylene terephthalate plates were used to cast the film solution after making it in liquid form, and we chose these plates because of their ability to withstand the temperature within the oven.

### 2.2. Method

**Figure 2:**
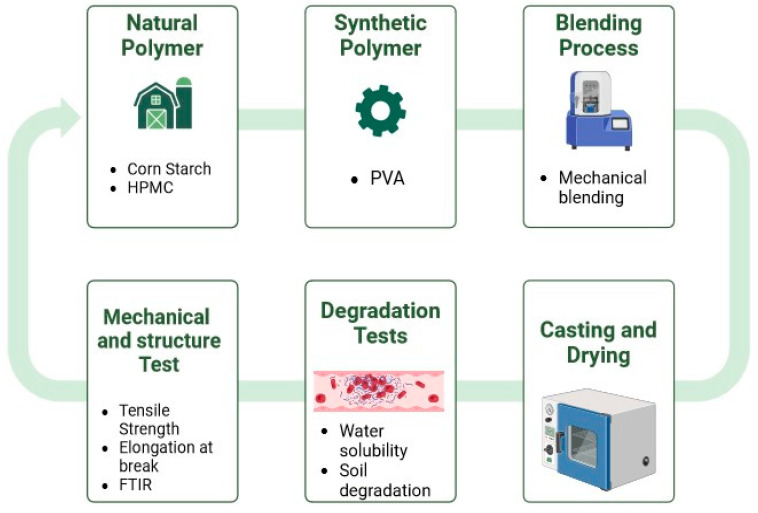
Flow chart of preparation of the composite films and the types of analytical tests that were performed.

Figure 2 summarizes the experimental procedures of polymer blending between synthetic polyvinyl alcohol (PVA) with natural polymers, namely corn starch (CS) and hydroxypropyl methylcellulose (HPMC), to produce unique polymers with outstanding properties that combine the properties of both polymers.

#### 2.2.1. Preparation of Polyvinyl Alcohol (PVA)

The PVA solution was prepared by mixing 3 g of PVA powder with 50 mL of warm distilled water at 200 rpm. The mixture temperature was gradually increased up to 80 °C until it achieved complete homogeneity.

#### 2.2.2. Preparation of Corn Starch-Polyvinyl Alcohol Blend Films (Csp)

**Corn starch and polyvinyl alcohol** blend films were prepared using a solvent-casting technique, as 3 g of powdered corn starch was mixed with 50 mL of deionized water. Glycerol was used as a plasticizer to enhance the elasticity, facilitate molding and shaping, and lessen the surface friction; the glycerol was added constantly at 500 rpm for 30 min. The temperature gradually increased from 40 to 95 °C while stirring to enhance starch gelatinization. PVA was added to different amounts of the gelatinized starch by stirring at 95 °C and 500 rpm for 30 min to ensure complete mixing. The different solutions were cast on polyethylene terephthalate (PET) plates and dried in an oven for 24 h at 40 °C, forming a film of the polymer blend. Table 1 lists the compositions of the different corn starch–polyvinyl alcohol blends.

#### 2.2.3. Preparation of Hydroxypropyl Methylcellulose–Polyvinyl Alcohol (HPMCP) Blend Films

**Figure 3:**
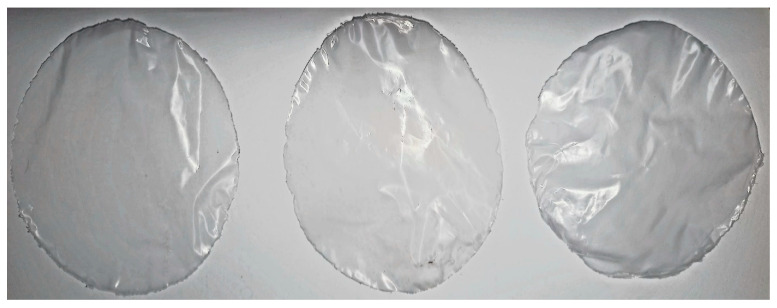
The sample shape for different blended films; the first sample on the left is pure PVA, and then CS/PVA and HPMC/PVA are on the right.

**Figure 4:**
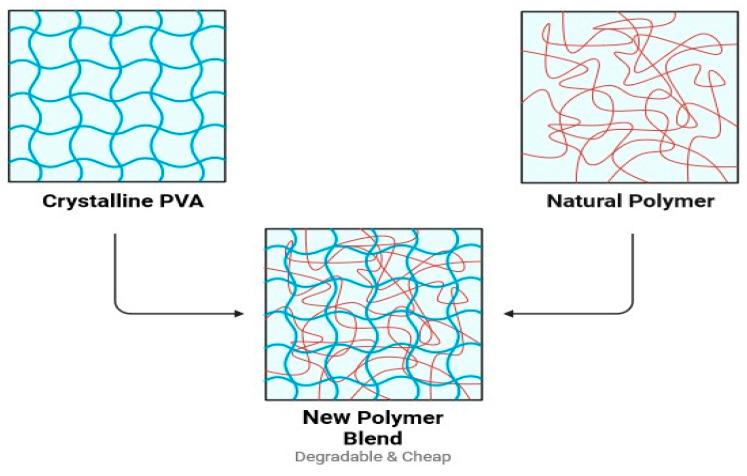
The final hypothetical design of the new polymer’s intermolecular structure after blending the two polymers.

HPMC with PVA blend films were prepared by adding 50 mL of deionized water to 0.5 g of dry powder HPMC in the presence of 1 gm glycerol as a plasticizer. For approximately 10 min, the HPMC solution and glycerol were mixed at 500 rpm, raising the temperature reaction of HPMC gradually from 40 to 75 °C. PVA was added to various amounts of HPMC solution at 75 °C for 10 min to ensure complete gelatinization. The different prepared mixtures were poured on polyethylene terephthalate (PET) plates and dried in an oven for 24 h at 40 °C, forming a film of the polymer blend. Table 2 lists the compositions of different HPMC and PVA blends. Figure 3 shows the final shape of the produced polymers including pure PVA and its blends. It is obvious that the three samples are similar in transparency, and no opacity is observed. Figure 4 is a hypothetical diagram that depicts the final polymer intermolecular structure after blending the two polymers.

#### 2.2.4. Characterization of Blend Films Made of Csp and HPMCP

The surface morphology (SEM), tensile strength, elongation at break, water solubility, biodegradability of the film samples by soil burial test, Fourier-transform infrared spectroscopy (FTIR), and diffraction scanning calorimetry were examined to investigate the properties of the composite films.

##### Surface Morphology

The surface morphologies of the composite films were examined using a scanning electron microscope (SEM, JSM-IT200 Series, Joel company, France). Before sputter coating, the film sample was placed on a stub and air-dried. To increase the conductivity of the dried film sample, gold-palladium was sputtered over it under a vacuum. Subsequently, the coated sample was examined under an SEM with a magnification of 500× and an accelerating voltage of 20 Kv. 

##### Mechanical Properties

The mechanical properties (tensile strength (TS) and % elongation at break) were measured according to ASTM D882 *Standard Test Method for Tensile Properties of Thin Plastic Sheeting*, using a Zwick/Roell tensile testing machine in polyethylene laboratories (Sidi Kerir Petrochemicals Company, Alexandria, Egypt) [22]. The measurement was performed by placing each film with a standard size of 2.54 × 15 cm into the grip; 50 mm and 50 mm/min were chosen as the beginning grip spacing and crosshead speed, respectively, to reach the maximum load of 500 N. This test was performed for each film sample, and the average of the three measurements was taken as the result at ambient temperature (25 °C).

##### Water Solubility (WS) Test

The WS of the films was examined using the methodology outlined by Rhim et al. [23] and Patil et al. [24]. A 5 × 5 cm specimen of each type of film sample was weighed and placed in a beaker containing 50 mL of distilled water for different times (1 and 6 h) in a controlled environment chamber (65% RH, 25 °C) wrapped in aluminum foil. After the required time, the submerged film pieces were removed from the beakers, dried, and weighed to investigate the final weight of the sample after degradation by water. 

##### Biodegradability of Blend Films

The biodegradability of the composite films was investigated using a soil burial test following the procedure described by Medina-Jaramillo et al. [25] and Patil et al. [24]. The test was performed in a transparent plastic box filled with soil; the film samples were cut into 2 × 2 cm pieces, weighed, and then buried in the soil at an ambient temperature of 27.5 °C and a relative humidity (RH) of 70.5%. Water was sprayed twice daily to maintain a constant soil hydration level. At periodic times (1 to 14 days), the film samples were removed by rinsing with water to remove adherent soil to calculate the dry weight of recovered samples to estimate the rate of film deterioration and the reduction in the weight. 

##### Fourier-Transform Infrared Spectroscopy (FTIR)

The FTIR analysis of the films was performed using an InfraRed Bruker Tensor 37 at the Central Laboratory unit in the Faculty of Science Alexandria University. The average true range (ATR) of the analysis, which was conducted at room temperature, was 700–4000 cm^−1^.

##### Diffraction Scanning Calorimetry (DSC)

DSC is a thermal analysis instrument that evaluates the heat flow and temperature of the phase transitions as a function of time and temperature. The measurements were performed using a NETZSCH DSC 214 Polyma DSC21400A-0257-L in the Sidi Kerir Petrochemicals Company laboratories by cutting the polymer film into multiple circular sections with a mass of approximately 10 mg in a temperature range of 0–250 °C in an inert gas (N_2_) atmosphere. The heating and cooling rates were 10 K/min.

## 3. Results and Discussion

### 3.1. Surface Morphology of Blend Films

**Figure 5:**
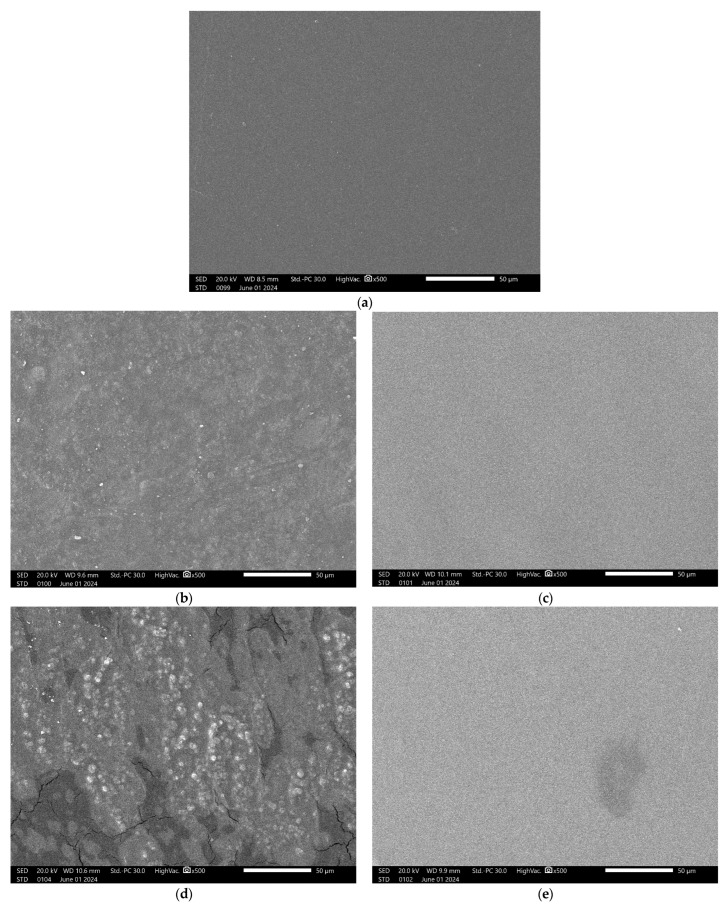
Surface morphology of (**a**) pure PVA, (**b**) Csp 70 composite film, (**c**) Csp 90 composite film, (**d**) HPMCP 420 composite film, and (**e**) HPMCP 540 composite film.

The surface morphology of PVA pure samples and composite films made of starch/PVA and HPMC/PVA were examined using 500× magnification scanning electron microscopy (SEM). For polymer blends, it is well known that obtaining the appropriate material properties critically depends on the morphological management of the corresponding phases [26]. Compared with the composite films, as shown in Figure 5a, no visible particles were scattered throughout the PVA matrix [27].The surface of the pure PVA film was smooth and flat, indicating the creation of a transparent and clear PVA film.

#### 3.1.1. Csp Blend Films

The SEM image of the PVA/St films showed fewer surface cracks, and starch particles seemed to be embedded in the matrix, which was due to the reorganization of polymer chains in the PVA/St matrix [28]. Starch and PVA are not completely miscible in Csp70, and the blend aggregates during the film-forming process. Ref. [29] A nonuniform phase is shown in Figure 5b. For Csp70, a few agglomerations, most likely made of non-gelatinized starch, were visible in the film, but the material’s integrity was unaffected. As a result, the blend’s three components (starch, PVA, and glycerol) were well-matched [30]. This is usually due to the two coexisting phases, namely the PVA-poor phase and the starch-rich phase, and a certain amount of starch polymer is immiscible [13]. Figure 5c shows the SEM picture of the CsP 90 film surface, which displays a homogeneous and smooth structure. This suggests that the structural integrity of the film’s structure was preserved even with an increase in PVA concentration. Our result of compatibility is in line with Patil et al. [24] and Tian et al. [31], who found that the starch polymer reverses to a dispersed phase from a continuous phase as the PVA level increases, indicating the miscibility of the amorphous part of starch with PVA. Strong bonding contacts were observed at the hazy interface between the two polymers. The CSP composite films exhibit improved mechanical performance, which aligns with the mechanical property data, owing to their superior starch–PVA compatibility.

#### 3.1.2. HPMC Blend Films

The surface morphology of HPMCP 420, as shown in Figure 5d, reveals a compact and nonhomogeneous surface structure. HPMC granules could be seen in the matrix, and the cracks in the surface are due to the high vacuum drying during sample preparation [32]. The greater light reflection from the sample surface causes its shiny appearance [33]. The HPMCP 540 film revealed fewer surface fractures, and HPMC particles appeared to be incorporated into the matrix, as shown in Figure 5e, because of the reorganization of PVA with the HPMC matrix. The SEM image of HPMCP 540 exhibited a homogenous composite film with a flat surface, demonstrating that the surface structure of the film improved, and the compatibility of the composite film increased with the increasing PVA content.

### 3.2. Mechanical Properties of Blend Films

The mechanical properties were measured to investigate the mechanical performance of samples and the effect of blending PVA with corn starch and HPMC polymers. 

#### 3.2.1. Csp Blend Films

**Figure 6:**
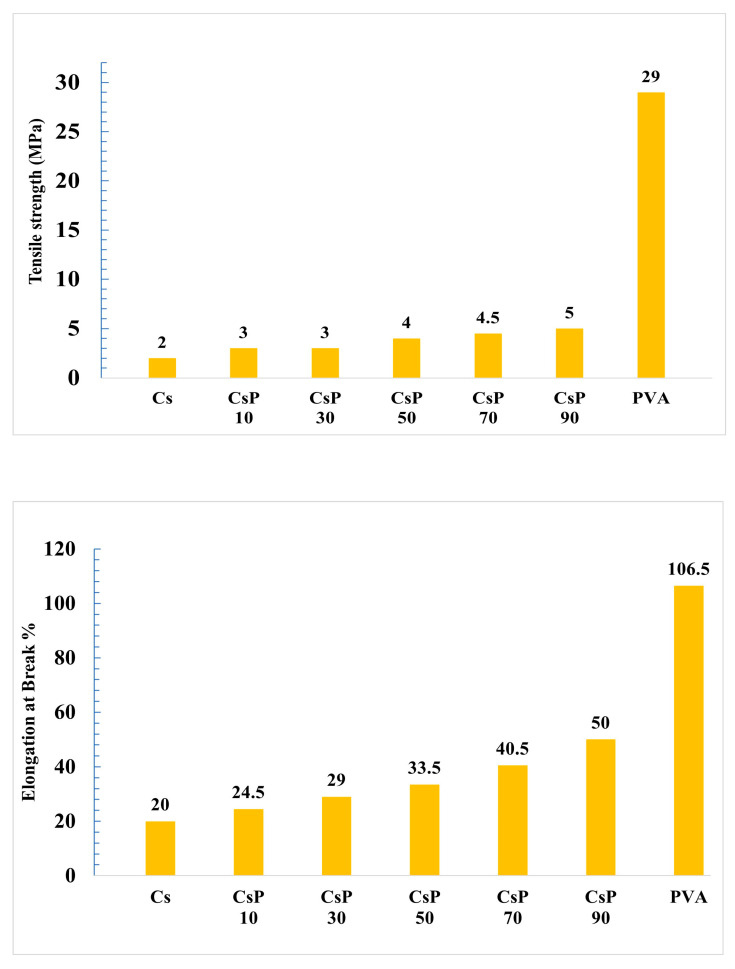
Tensile strength of PVA and starch/PVA blended films (**top**); elongation at break of PVA and starch/PVA blended films (**bottom**).

As Shown in Figure 6, the pure PVA films demonstrated the highest tensile strength (TS) of 29 mPa and 106.5% elongation at break, while pure starch films have poor properties since their TS is 2 mPa and their elongation at break is 20%, which indicates a large difference in tensile strength and elongation at break between PVA and starch. By increasing the integration of PVA from Csp 10 to Csp 90, there is a gradual improvement in TS and elongation at break as the highest TS values of blends were for Csp 90 film, with 5 mPa and a 50% elongation at break, while the lowest properties were for Csp10 with 3 mPa and an elongation at break of 24.5%, proving the high crystallinity of PVA and the amorphous characteristics of starch. The reason for the weak mechanical properties of corn starch is its containment of amylose content that usually has bigger crystalline domains [34], leading to greater gaps in the polymer structure [35] and, consequently, higher amorphous characteristics and a lower molecular weight. This results in the lower tensile strength, Young’s modulus, and elongation at break of the starch film. Our results are in line with the findings of Patil et al. [24], Ashraf et al. [36], and Phattarateera et al. [37], as these authors reported that as the concentration of PVA increased in the starch/PVA blend, the tensile characteristics of starch/PVA blended films were enhanced. The decrease in the mechanical properties, which occurred due to the presence of starch, limits the choice of blend used in applications that require high strength. 

#### 3.2.2. HPMC Blend Films

**Figure 7:**
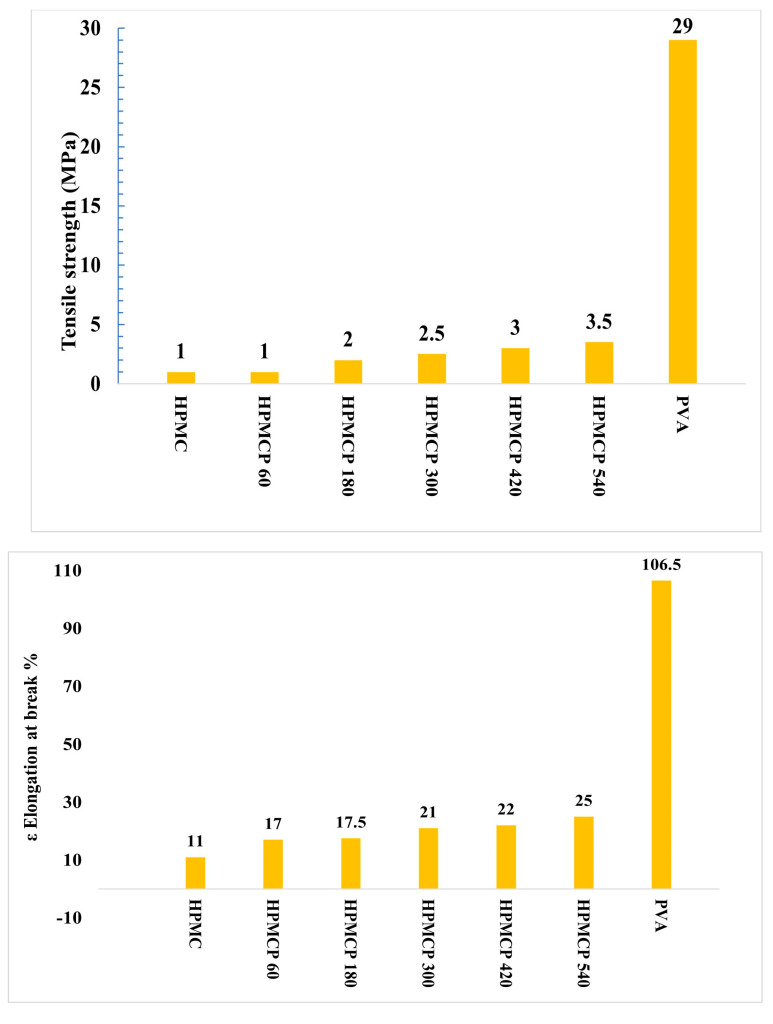
Tensile strength of PVA (**above**) and HPMC/PVA blended films. Elongation at break of PVA and HPMC/PVA blended films (**bottom**).

Pure HPMC showed a very poor TS of 1 mPa and elongation at the break of 11% compared to pure PVA, as shown in Figure 7, due to the high crystallinity of PVA and the amorphous characteristics of HPMC. With the increasing inclusion of PVA in the films, the values of TS and elongation at break % also increase, as HPMCP-540, which has the highest PVA concentration, has a TS of 3.5 mPa and an elongation at break of 25%, while HPMC60 has the lowest properties of 1 Mpa and an elongation at break of 17%. This increase in mechanical properties proves the compatibility of HPMC with PVA due to the hydrogen bonds between PVA and HPMC [38]. Owing to the good compatibility between starch and PVA and the stronger hydrogen bonding interactions between them in comparison to HPMC/PVA blended films, blended films made of PVA/starch have higher TS and elongation at break values than blended films made of PVA/HPMC. 

Theoretically, starch and PVA interact synergistically through hydrogen bonding, enhancing the functional qualities of materials made of starch [39]. There are two methods by which starch and PVA can form hydrogen bonds.

- Hydrogen bonds formed directly through hydroxyl groups [40].
- Water-mediated hydrogen bonding via water molecule plasticization [41]

The addition of PVA to cellulose in the film system significantly improved the functional characteristics because PVA and the hydroxyl groups on the cellulose molecular chains formed hydrogen bonds [42]. 

### 3.3. Water Solubility (WS) of Blended Films

The weight of the soluble materials remaining after immersion in water is indicated by a film’s water solubility (WS) as in Equation (1). The water solubility of the samples was analyzed to determine their interaction with the surrounding aquatic atmosphere, since all the ingredients, including HPMC, starch, PVA, and glycerol, have a hydrophilic nature due to the presence of hydroxyl groups in their structures; therefore, high-water solubility (WS) values in the film samples were expected. Equation (1) is as follows:

(1) $$Water\;Solubility=\frac{Initial\;weight\;dry-weight\;wet}{Initial\;weight\;dry}\times 100$$

where initial weight is the weight before immersing in water and weight wet is the weight after immersing in water.

#### 3.3.1. Csp Blended Films

**Figure 8:**
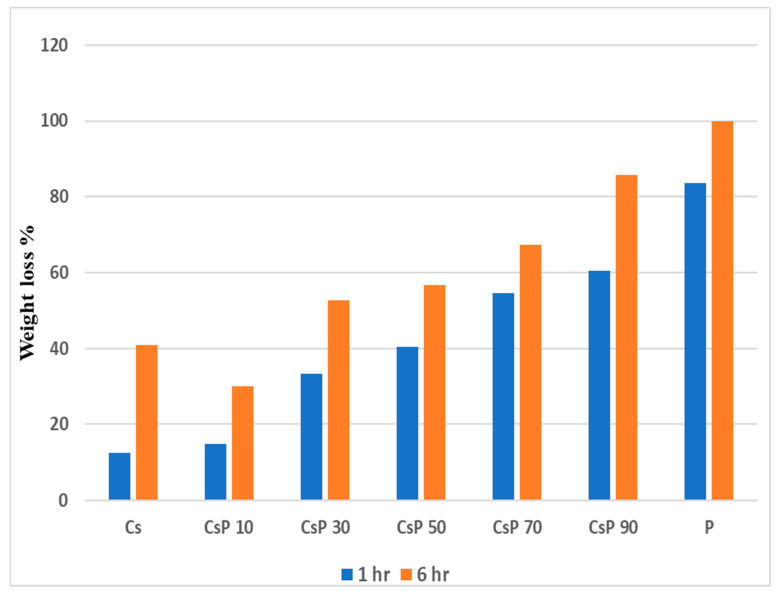
Weight loss of PVA and starch/PVA composite films after 1 h and after 6 h.

As shown in Figure 8, pure PVA exhibited a strong WS of 83.7% weight loss in 1 h and 100% weight loss in 6 h, making it practically entirely soluble in water due to its containment of a hydroxyl group that has a high degree of hydrophilicity. Pure corn starch shows the lowest WS of 12.5% weight loss in 1 h; starch has a low water solubility because of its molecular structure, since it is made up of long chains of glucose molecules, which form a complex and tightly packed structure, making it very difficult for water molecules to break down the starch molecules. In addition, the low concentration of hydrophilic groups, such as the hydroxyl group in the starch structure, weaken the water solubility of starch [43]. The water solubility increased gradually from 15.4 to 60.4% when increasing the PVA amount added to the starch, as in samples from 10 to 90% (samples Csp10 to Csp 90, respectively) because of the perfect homogeneity between PVA and starch and the increasing concentration of hydroxyl groups from PVA in the blend, leading to the increase in the water solubility of the composite films [44,45]. After 6 h of immersion in water, the weight loss of samples increased due to the greater water permeability of the intermolecular structure of the blend, as shown in Figure 7.

Our findings are also in line with those of other investigations by Patil et al. [24], as a similar pattern was observed in blends of potato starch and PVA, where the higher PVA and hydroxyl groups led to more hydrophilic films and high WS. Taghizadeh et al. [46] and Cano et al. [13] concluded that the greater the starch quantity in the Starch/PVA composite films, the more resistance of film″ water solubility and low water solubility. Abral et al. [47] and Savadekar et al. [48] found that moisture absorption properties decreased when starch was added to PVA.

#### 3.3.2. HPMCP Blended Films

**Figure 9:**
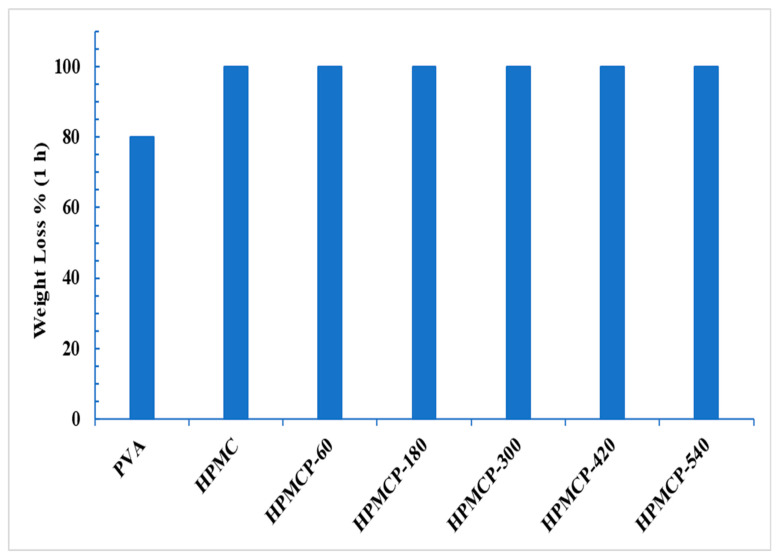
Weight loss of PVA blank film HPMC/HPMCP composite films after 1 h.

As shown in Figure 9, all samples were completely solubilized after 1 h in water, whereas the blank sample (PVA) experienced a weight loss of 83.7% after 1 h, indicating that the HPMC had a higher water solubility value; this is because HPMC contains many hydrophilic hydroxyl groups.

The high solubility of HPMC can be attributed to the presence of more of the three hydroxyl groups, which makes HPMC extremely hydrophilic and highly soluble in water [49]. Guirguis O et al. [50] found that the hydrophilicity degree of HPMC was very high in a blend of HPMC/PVA. Our results are consistent with Ghadermazi R et al. [51], who concluded that the formation of hydrogen bonds within the polymer chains and hydrophilic plasticizers, such as glycerol, could also increase the moisture content of films by creating more space between the chains for water absorption. 

### 3.4. Biodegradability of Polymer Blend Films

The ability of the films to undergo biodegradation through soil burial tests was studied as part of the environmental impact assessment of biodegradation analysis of the films. Biodegradation is the chemical dissolution or disintegration of materials caused by the enzymatic activity of living organisms, such as bacteria, yeast, and fungi. The soil burial test is an outdoor experiment that offers a realistic setting in the presence of humidity and different microbe types. To prevent the impact of the film‘s shape on its biodegradability, every specimen used in the tests was the same size and shape. The soil burial test was performed using the method performed by Medina-Jaramillo et al. [25]. To conduct the biodegradation experiment, soil analysis was performed at the organic agriculture and soil fertility laboratory of the Egyptian Ministry of Agriculture and Land Reclamation. Table 3 shows the soil specifications used in the soil burial test. Equation (2) determines the biodegradation equation, as follows:

(2) $$Biodegradation\;(Weight\;loss\;\%)=\;\frac{Initial\;weight-weight\;after\;burying}{Initial\;weight}\times 100$$

The biodegradability of the pure HPMC, pure starch, pure PVA, HPMC/PVA blend, and starch/PVA blend films was investigated using a soil burial test under ambient conditions.

#### 3.4.1. Csp Blend Films

**Figure 10:**
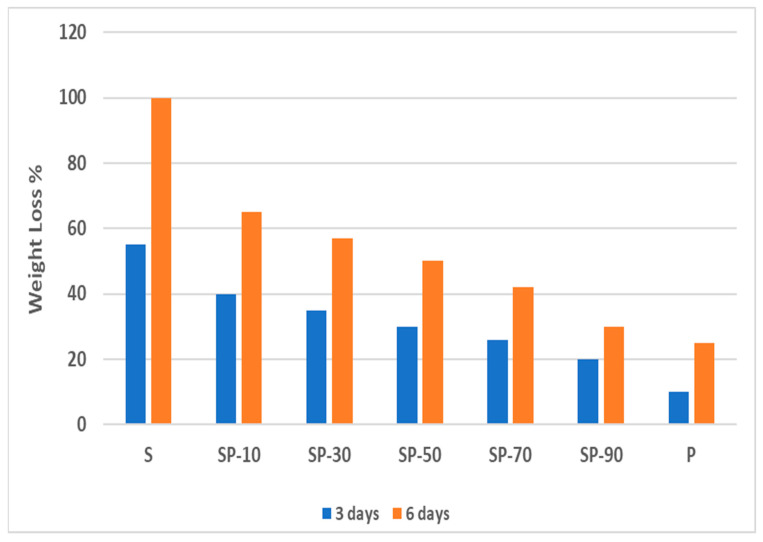
The pure PVA and blended starch films.

It has been found that every film sample decomposed in the soil and lost a significant amount of weight. Visual observations revealed that PVA shows greater resistance to deterioration from soil burial as the PVA weight loss was only 10 and 25% after 3 and 6 days of soil burial, respectively [35]. The low biodegradation properties of PVA are due to the highly crystalline structure of PVA and the lack of *Pseudomonas 0–3*, a bacterium that completes polymer breakdown, in agricultural soil [52]. The pure starch films have a biodegradation rate higher than PVA, as after 3 days of burial a starch film, the weight loss was 55%, reaching 100% after 6 days of burial. The high biodegradation properties of starch are because of its amorphous structure, which contains polysaccharides that are required for soil and many degrading bacteria inside the soil. Figure 10 illustrates that the samples from Csp 10 to Csp 90 degraded in a shorter period than the pure PVA. As the PVA % increases in the sample, the biodegradation rate lessens; this is why the Csp90 composite film samples exhibited the lowest weight reduction among the blend samples. This result is owing to the fact that incorporation of a high amount of cornstarch in the PVA molecular structure causes weaknesses in these intermolecular forces of PVA bonds.

The degradation rate of the Csp composite films was enhanced after 6 days because the water in the soil diffused in higher amounts and more rapidly through the blend samples and made the bacteria more active in biodegrading the composite films. In addition, the compatibility of the blended films of starch/PVA, the exposure to higher moisture content in the soil, and the highly hydrophilic nature of starch/PVA composite films led to more water absorption and rapid bacterial diffusion, resulting in an increased biodegradability rate.

For the burial period of 12 days, the blended starch/PVA samples were completely degraded after 12 days.

The biodegradation investigations of starch/PVA blend films conducted by Negim et al. [15] and Patil et al. [24] provide strong support for our findings, since they found that the starch/PVA films were completely broken down in 10–14 days in moist soil. In conclusion, the blend of PVA/starch has a biodegradability property better than pure PVA or pure starch, since our results show that adding starch to PVA increases the deterioration rate of PVA, allowing the creation of an environmentally friendly polymer type.

#### 3.4.2. HPMCP Composite Films

After only three days, the visual assessment revealed that the HPMCP films completely vanished in the soil. The samples of pure HPMC and HPMC/PVA composite films deteriorated very quickly in soil, in comparison to pure PVA and starch/PVA composite films. This is because of their very high hydrophilicity, which caused water in the soil to diffuse quickly into the film to swell and allow microorganisms to grow on the film, leading to the weight loss and disruption of the samples. The degradation occurred rapidly in the soil and produces a remaining small fragment of the soil with no visible evidence of any film fragments. After completing the biodegradation test, we performed an elemental analysis of the soil and observed that the biodegradation process increased the organic carbon content of the soil by 2%, and the total nitrogen content increased from 105 to 115 ppm by 9.5% while maintaining the PH of the soil at the same value, which increases the soil fertility and improves plant growth.

### 3.5. Fourier-Transform Infrared Spectroscopy

**Figure 11:**
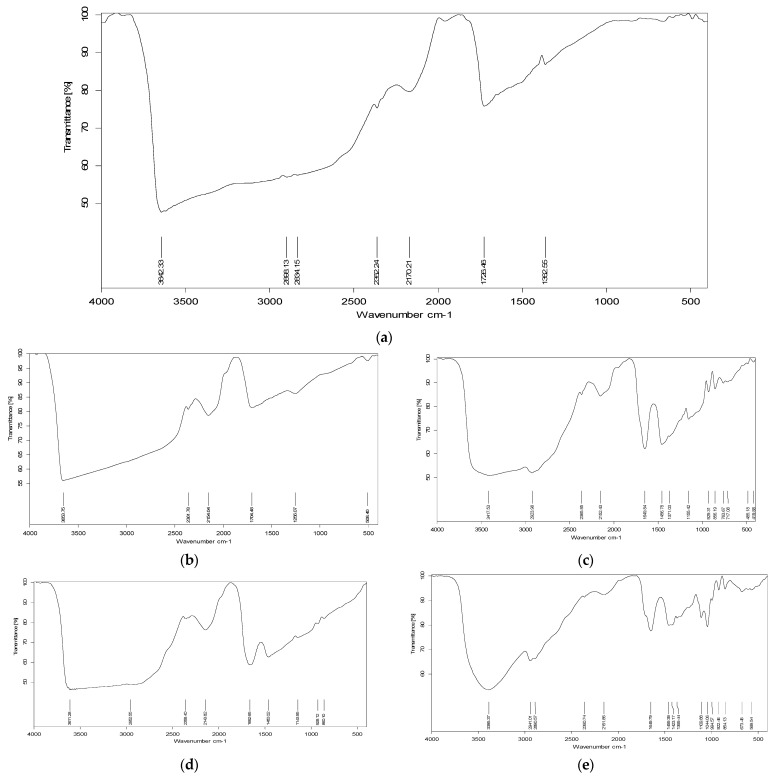
FTIR graphs. (**a**) VA, (**b**) CSP 70, (**c**) CSP 90, (**d**) HPMCP420, and (**e**) HPMCP-540.

FTIR spectroscopy was employed to examine the physicochemical composition and interactions between PVA, blended starch, and blended hydroxypropyl methylcellulose composite films in the 4200–200 cm^−1^. As shown in Figure 11, pure PVA has noticeable peaks that most closely match the hydroxyl and acetate groups. The existence of both intramolecular and intermolecular -OH hydrogen bonds was related to the broader band [53] at 3642.33 cm^–1^. The alkyl group C–H bond vibrational stretching was shown by the peaks between 2898 and 2834 cm^−1^. The carbonyl (C=O) group of the remaining PVA acetate group is responsible for the band at 1726.46 cm^−1^. The signal at 1362.55 cm^−1^ indicated the presence of hydrocarbon (alkane) in PVA.

The incorporation of corn starch in the PVA resulted in peak modifications, since the starch/PVA samples exhibited absorption peaks in the region of 3600 cm^−1^, which were associated with the vibrations from the stretching of the -OH groups. The presence of the C–H group in starch and PVA caused a band to appear at wavenumber 2923.98 cm^−1^. The peak exhibits the bending vibration of the -OH group’s hydrogen bonding at 1704.48 cm^−1^. At 1640 cm^−1^, on the other hand, the typical hydrogen bonding bending vibration was noted [54]. It is interesting to note that the peaks at 1640 cm^−1^ were noticeably different because of the -OH vibration in the water’s scissors mode compared to the hydration in starch [55,56]. The C-O stretched at 1155.42 cm^−1^, proving the complete homogeneity of the starch with PVA polymer. When the amount of PVA in the composite films was increased, the peak intensity of the C-O bonds in the C-O-C groups also increased. The strong hydrogen bonds between the PVA and cornstarch components were visible in the FTIR spectra of the composite films since both polymers have many -OH and C-O groups that can generate a network of hydrogen bonds, increase their compatibility, and improve the quality of the composite. Our results are in line with those of Patil et al. [24], Tian et al. [31], and Negim et al. [15]. 

The blending of the two polymers (PVA/HPMC) modified the peaks that characterized the PVA spectra. The composite film samples exhibited absorption peaks in the 3611 cm^−1^ region that are linked to vibrations resulting from the stretching of the -OH groups in the HPMC–PVA mixture. A band at wavenumber 2941 cm^−1^ was observed in HPMC and PVA due to the presence of the C–H group. At 1650 cm^−1^, the peak displayed the bending vibration of the C=O groups, and further evidence of the mixing of HPMC and PVA was provided by C-O stretching at 1109.88 cm^−1^. The peak C-O bond intensity in the C-O-C group increased with the increasing PVA content in the composite films. Because both polymers contain many -OH and C-O groups, a network of hydrogen bonds may be formed between them, increasing their compatibility and enhancing the properties of the composite. Our results are in line with those of Mahesh et al. [57] and Bhajantri R et al. [58].

### 3.6. Thermal Properties of Polymer Blends

**Figure 12:**
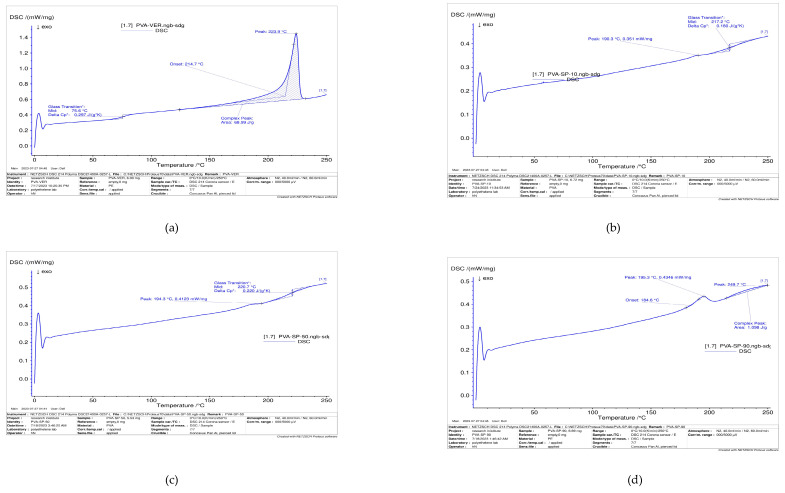

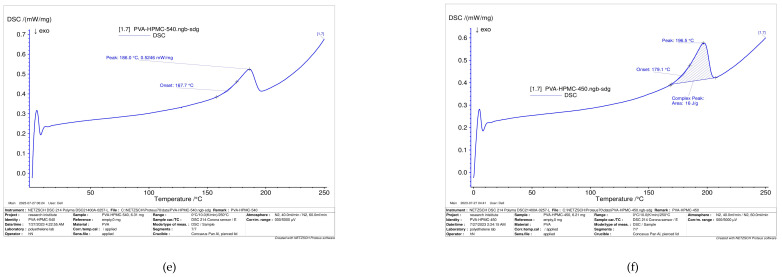
Thermal analysis of (**a**) pure PVA, (**b**) Csp 10 composite films, (**c**) Csp 70 composite films, (**d**) Csp 90 composite films, (**e**) HPMCP 420 composite films, and (**f**) HPMCP 540 composite films.

The durability of plastic films under extreme heating conditions determines their usefulness for numerous technical and industrial applications. Differential scanning calorimetry (DSC) was used to maintain and enhance the quality of the plastic materials. To determine how temperature affects plastic quality in various plastic applications, a thorough investigation must be carried out. The weaker thermal stability of biopolymers, particularly natural polymers, compared with petroleum-based polymers, is one of the main reasons for their limited application in the packaging sector [59,60]. As shown in Figure 12, the pure blank PVA film had the lowest glass transition temperature (75.6 °C) in comparison to blended films and the highest melting temperature (223.9 °C). The low molecular weight of the PVA backbone and the use of glycerol as a plasticizer are the reasons behind PVA blank film’s lower T_g_ [61]. Wu et al. [62], showed that plasticizers can penetrate PVA macromolecules and disrupt their structural regularity, which would lower the PVA melting point and reduce crystallization. As shown in Table 4 [63], some factors affect the glass transition temperature of plastic films (T_g_).

One of the most important factors in the production of plastics is their melting points. The temperature at which plastic must melt to generate a variety of plastic items is specified. As shown in Table 5 [60], some factors affect the melting temperature of plastic films. For blank films, PVA has the highest T_m_, making it need a high temperature to remelt if wants to be recycled again.

Poly(vinyl alcohol) is a linear aliphatic polymer with secondary hydroxyl groups on each carbon atom. The concentration of hydroxyl groups significantly affected T_g_ and T_m_. Generally, any structural features that limit segmental mobility or free volume increase T_g_ [64]. The PVA matrix limited segmental movement and increased the T_g_ of the blended films, and the addition of starch powder increased the T_g_ peak. In this scenario, as shown in Figure 12b–d and Table 6, for Csp 10, Csp 70, and Csp 90, as the amount of PVA in the blend increased, the temperature of the composite films shifted to a high glass transition temperature from 190.3 to 195.3 °C. The inclusion of starch in the PVA matrix increased the number of hydroxyl groups and hydrogen bonding, which increased the T_g_ peak, because both PVA and starch are polar polymers containing hydroxyl groups [65], causing the T_g_ to shift to higher values due to the increase in the addition of PVA. The different composite films also shift to a high T_g_ owing to the increasing number of connections between the composite films, which makes the composite films more brittle.

As shown in Figure 12, the peaks of CSP 10 and CSP 70 are more symmetrical than those of CSP 90. The peaks of CSP 10 and CSP 70 had a more uniform distribution. Therefore, the symmetry of the peaks may be related to the uniformity of the distribution of starch with PVA [17]. 

As shown in Figure 12b–d, the melting temperature of the composite films (Csp 10, Csp 70, Csp 90) increased from 217.2 to 221 °C due to the fact that the extra-cyclic hydroxyl group of starch and the hydroxyl group of PVA produced strong hydrogen bonds, which gave the blends a higher degree of energy stability. This is why the Tm values for blended films increase as the amount of PVA increases [66]. Owing to their smaller size compared to the polymer molecules, the plasticizer molecules (glycerol) could pass through the polymer matrix. Consequently, the adhesive interactions between PVA and starch molecules were replaced by polar attractive forces between the plasticizer and chain segments, breaking the granular and crystalline structures of the starch/PVA mixes. Consequently, one could anticipate a decrease in T_m_ [67]. Our results are in line with those of Sreekumar et al. [68] who found that the melting and crystallization enthalpies decreased and the melting temperature decreased as the starch content of the blend increased.

As shown in Figure 12e,f and Table 6 for HPMCP-420 and HPMCP-540, it was observed that when the amount of PVA was increased, the glass transition temperature also increased from 167.7 to 179.1 °C, respectively, due to the intermolecular interaction, which occurs between the HPMC/PVA composite films. It was observed that the T_g_ of the composite films increased, but to a lesser extent than that of the starch/PVA composite films; in addition, the melt temperature of the HPMCP composite films shifted to a higher temperature, i.e., from 168 to 196.5 °C, but this was less than for the corn starch/PVA composite films because when HPMC and PVA composites were cast from aqueous solution, they were incompatible at all concentration levels [69], and HPMCP contained a high amount of free water particles in the composite films in comparison to the corn starch/PVA composite films, which caused composite films to lose their free water particles rapidly.

To sum up, increasing T_g_ after adding natural polymers to PVA indicates that molecular weight and intermolecular interactions between blend molecules are improved. However, Tm decreases after adding a natural polymer, making the blend require a lower temperature to remelt and be recycled again, and this is due to the amorphous structure of the natural polymer that decreases the crystallinity of the polymer blend.

**Figure 13:**
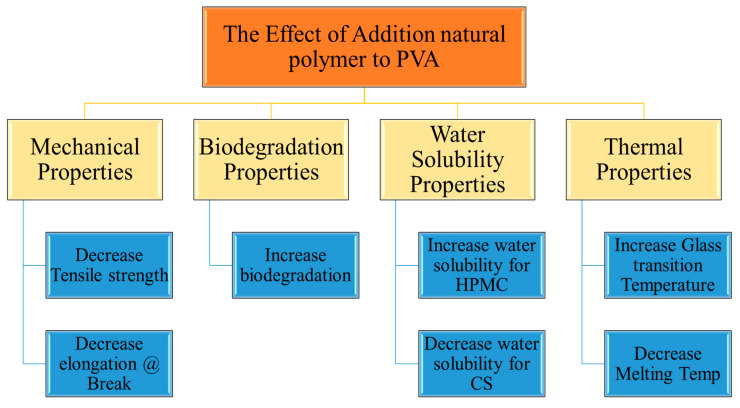
The effect of adding natural polymers on the PVA properties.

Figure 13 summarizes the effect of addition of natural polymers, namely corn starch (CS) and hydroxypropyl methylcellulose (HPMC), to polyvinyl alcohol, and the impact on the mechanical, biodegradation, water solubility, and thermal properties of PVA. As seen in the figure, there are some similarities between the effect of adding CS and HPMC on the PVA properties. Both CS and HPMC decrease the mechanical properties, decrease the melting temperature T_m_, increase the T_g_, and improve biodegradation. However, they act dissimilarly in terms of the water solubility, as HPMC enhances the water solubility and CS slows it down.

## 4. The Proposed Applications of the New Blends

**Figure 14:**
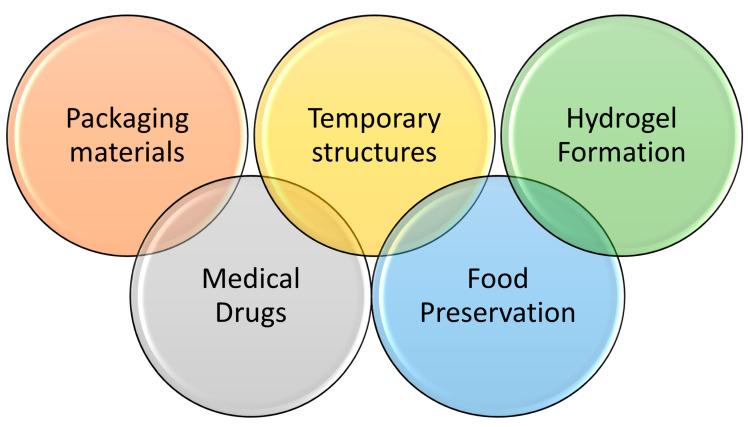
Proposed applications for starch/PVA and HPMC/PVA blended films.

The research goal is to produce biodegradable films using inexpensive natural polymers combined with a specially developed commercially modified biodegradable polymer to minimize the cost of the final product and maximize its degradation rate, making it competitive with the industrial polymers that are currently in use daily. To enhance our research output, we proposed many applications that match the produced polymers’ properties, as some applications can be applicable for the PVA/starch blend and not valid for the PVA/cellulose blend. Figure 14 outlines the proposed applications for the prepared polymer blends, while Table 7 explains the feature of each blend for an expected application and the reason for choosing that application.

## 5. Future Directions for Advancing Biodegradable Polymer Industries

While substantial progress has been made in developing biodegradable polymers by blending polyvinyl alcohol (PVA) with natural polymers, opportunities abound to further refine these materials for widespread commercial adoption and to compensate for the deficit in some properties of the blend. The following strategies or directions highlight potential avenues for enhancing the properties, applicability, and environmental benefits of the produced polymer blend.

**Diversifying plasticizer use:** Exploring a broader array of plasticizers, including sorbitol, citric acid, succinic acid, malic acid, tartaric acid, and combinations, such as glycerol/urea mixtures, could significantly improve the mechanical and thermal characteristics of the films. Such diversification aims to optimize the compatibility between PVA and natural polymers, like starch or HPMC, potentially unlocking new applications and performance enhancements.

**Incorporating organic oils:** Integrating various organic oils, such as used cooking oil, into PVA blends offers the dual benefit of enhancing film biodegradability while also allowing for antimicrobial properties. The inclusion of specific essential oils, like oregano oil, could further augment the antimicrobial efficacy of the films, presenting a value-added feature for applications requiring sterility.

**Utilizing calcium carbonate (CaCO_3_):** Leveraging the affordability and abundance of calcium carbonate as a filler could strengthen the mechanical properties of the polymer films. Given its nontoxicity and role in soil pH neutralization, CaCO_3_ incorporation also promises to improve the environmental footprint of the biodegradable films upon degradation, positively contributing to soil health.

**Exploring smart packaging solutions:** The advent of intelligent packaging technologies heralds a new era for the packaging industry. Biodegradable films integrated with smart functionalities, such as freshness indicators or temperature sensors, could revolutionize food safety and shelf-life monitoring. Additionally, the development of lignocellulosic biomass-based composites offers innovative, eco-friendly options for packaging that align with consumer demand for sustainable solutions.

By pursuing these future directions, the field can address the remaining challenges and unlock the full potential of biodegradable polymers. Such advancements not only contribute to the sustainability of the materials sector but also offer promising commercial opportunities in packaging, agriculture, healthcare, and beyond, heralding a new chapter in the evolution of environmentally friendly materials.

## 6. Conclusions

The pervasive use of non-biodegradable single-use plastics, primarily composed of polyethylene and polypropylene, poses a formidable threat to our environment and health, as they accumulate in ecosystems for centuries due to their persistent nature. Addressing this critical issue, our study explores the integration of cost-effective, natural polymers—starch and hydroxypropyl methylcellulose (HPMC)—with polyvinyl alcohol (PVA) to engineer biodegradable alternatives capable of competing with conventional single-use plastics as the process of polymer blending is cost-effective and easy to apply. Through detailed experimentation and a variety of characterization techniques, we have developed two novel types of polymer blends that exhibit promising solubility, biodegradability, and thermal properties.

The main disadvantages of PVA are its high cost and slow rate of biodegradation. To eliminate these disadvantages, we produced two different types of degradable polymers using a blend of polyvinyl alcohol (PVA) with two types of cheap natural polymers to produce cheap blends. The two types of natural polymers are corn starch (CS) and hydroxy methyl cellulose (HPMC). Our findings illuminate the path toward mitigating the environmental impact of plastic waste, demonstrating that blending PVA with natural polymers not only improves its biodegradability but also maintains acceptable mechanical integrity. The surface structure investigation using a scanning electron microscope (SEM, JSM-IT200 Series) finds that there is a complete homogeneity between the PVA and the natural polymer in high percentage of PVA, but surface cracks and natural polymers appear in low percentages of PVA, as the surface was homogenous in Csp 90 and HPMC 540, while few cracks exist in HPMC 420 and Csp 70. The addition of starch was found to modulate the biodegradation properties of PVA in soil, as the addition of 50 w/w of starch can duplicate the rate of PVA biodegradation, whereas CS addition reduces the water solubility of pure PVA. HPMC addition notably accelerated the biodegradation process and increased the water solubility since the addition of any amount of HPMC accelerated the biodegradation of pure PVA from 10 to 100% in 3 days and increased the water solubility from 80 to 100% in 1 h, underscoring the tailored functionality of these blends for diverse applications. Fourier-transform infrared (FTIR) spectroscopy further elucidated the molecular interactions and homogeneity within the blends, affirming the successful amalgamation of the polymers. The thermal analysis applied on pure PVA and its blends with natural polymers proves the complete homogeneity of blends and the improvement in thermal properties after adding natural polymers, as the increase in T_g_ refers to the enhancement of intermolecular forces between PVA and natural polymer molecules, while the decrease in T_m_ relates to the improvement of recycle prose as the blend requires a lower Tm to remelt. The results of mechanical properties investigation show a noticeable decrease in the mechanical properties of pure PVA after the natural polymers, which is an expected outcome as the biodegradation of the polymer and its mechanical properties have an inverse relation, and this matches with findings. The tensile strength declined from 29 for pure PVA to 5 MPa for Csp 90 and 3.5 MPa for HPMC 540; also, the elongation at break shrank from 106.5 to 50 and 25% for Csp 90 and HPMC 540, respectively. This research marks a significant stride towards the realization of sustainable, eco-friendly materials, offering a viable alternative to the detrimental single-use plastics that dominate our daily lives. By harnessing the inherent properties of natural polymers to enhance the performance of PVA, we present a compelling case for the potential of these blends to contribute to a more sustainable future. Our study not only provides a foundation for further exploration of biodegradable polymers but also exemplifies the interdisciplinary innovation that is critical for overcoming environmental challenges.

## Figures and Tables

**Table 1:** Compositions of different CS and PVA blends.

Sample Code	Starch (Cs) (g)	PVA (P) (g)	PVA *w*/*w*	Glycerol (g)
Cs	3	-	-	1
Csp 10	3	0.3	10	1
Csp 30	3	0.9	30	1
Csp 50	3	1.5	50	1
Csp 70	3	2.1	70	1
Csp 90	3	2.7	90	1
PVA	0	3	100	1

**Table 2:** Composition of different HMPC and PVA blends.

Sample Code	HPMC (g)	PVA (P) (g)	PVA *w*/*w*	Glycerol (g)
HPMC	0.5	-	-	1
HPMCP 60	0.5	0.3	60	1
HPMCP 180	0.5	0.9	180	1
HPMCP 300	0.5	1.5	300	1
HPMCP 420	0.5	2.1	420	1
HPMCP 540	0.5	2.7	540	1
PVA	-	3	100%	1

**Table 3:** The soil specifications used in the soil burial test before burying the samples.

Organic Material %	Main Elements (ppm)		Anions (ppm)				Cations (ppm)				Electrical Conductivity		PH
0.5	N	P	SO_4_	HCO_3_	CO_3_	Cl	Ca	Mg	K	Na	P.P.M	Us/cm	7.67
	105	0.9	19.4	183	--	106.5	28	7.2	34.7	23	106.2	166	

**Table 4:** Factors that affect the glass transition temperature for plastic films (T_g_) [63].

Factors	Effects	Relation
**Chain flexibility**	The nature of the polymer backbone and the groups that are directly connected to it define the intrinsic chain flexibility. While ringed structures cause the chain to stiffen, the aliphatic C-C and C-O bonds exhibit remarkable flexibility. The glass transition temperature rises when the chain becomes stiffer.	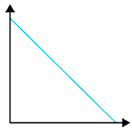
**Intermolecular interactions**	Intermolecular interactions and secondary bonding, such as hydrogen bonding, van der Waals force, induction forces, and dipole–dipole interactions, also have an impact on segmental rotations. These kinds of interactions raise the glass transition temperature because they make polymeric materials stiffer.	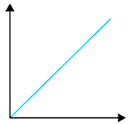
**Molecular weight**	The glass transition temperature of a polymer decreases with increasing molecular weight, and it is logical to assume that this temperature increases linearly with increasing chain-end concentration. On the other hand, we can say that this temperature decreases linearly as the molecular weight of the polymer increases.	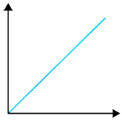
**Crosslinking**	The polymer becomes rigid and restricted due to crosslinking, which raises the glass transition temperature.	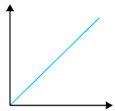
**Plasticizer**	Plasticizers separate polymer chains, reduce cohesive forces, and generally promote molecular mobility when added to polymers. The glass transition temperature is lowered, and the polymer brittleness is decreased by the plasticizer.	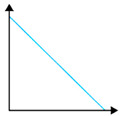

**Table 5:** Factors that affect the melting temperature for plastic films (T_m_) [60].

**The chemical structure of the polymer**	The melting temperatures of various plastics will vary due to their distinct chemical structures. For example, plastics with more hydrocarbon groups often melt at a higher temperature than plastics with various functional groups.
**Degree of crystallinity**	The melting point of crystalline polymers is higher than that of amorphous plastics. This is because crystalline plastics have molecules arranged in a certain arrangement that makes them more resilient to disintegration.
**The mass ratio of components in the plastic**	The mass ratio of a plastic’s constituent parts can also affect the plastic’s melting point.
**Additives**	The plastic’s melting temperature may change if additives are added. For example, thermal stabilizers can be added to increase the plastic’s melting temperature.

**Table 6:** Glass transition temperature T_g_ and melting temperature T_m_ for CS and HPMC/PVA blended films.

Sample	T_g_ (°C)	T_m_ (°C)
**PVA**	75.6	223.9
**CSP10**	190.3	217.2
**CSP70**	194.3	220.7
**CSP90**	195.3	221
**HPMCP 420**	167.7	168
**HPMCP 540**	179.1	196.5

**Table 7:** Expected application for starch/PVA and HPMC/PVA blended films.

Application	Types of Blended Films Used	Reason
***Packaging Materials*** **(single-use plastic)**	The blend of starch/PVA can used as a biodegradable film that can be employed in packaging and does not need high strength.	Biodegradable Good water solubility Good tensile strength
**Medical drugs**	Most innovative tablet ingredients are based on HPMC and other water-soluble cellulose derivatives because HPMC capsules breaking down in water or gastric juices at 37 °C were said to take just a little longer than gelatin capsules [34]. HPMC can be safely removed from the human body in a fair amount of time; the biodegradability of its cellulose backbone and nontoxic nature make it suitable for certain applications, like drug delivery [49].	Natural Nontoxic Biodegradable
**Temporary Structures**	For tissue engineering, it is possible to make scaffolds that are biodegradable and will eventually break down as the tissue regenerates.	High water solubility Biodegradable Environmentally friendly
**Food preservation**	The blend of HPMC/PVA can used in applications for food preservation and used as a natural film for monitoring the freshness of food [70].	Natural Biodegradable Nontoxic Environmentally friendly
**Hydrogel Formation**	It is possible to make use of the polymer’s high solubility in water to create hydrogels, materials with a high capacity to absorb and hold water.	Natural Nontoxic Biodegradable

## Data Availability

The original contributions presented in the study are included in the article, further inquiries can be directed to the corresponding author/s.
